# Water Soldier *Stratiotes aloides* L.—Forgotten Famine Plant With Unique Composition and Antioxidant Properties

**DOI:** 10.3390/molecules25215065

**Published:** 2020-10-31

**Authors:** Urszula Gawlik-Dziki, Piotr Sugier, Dariusz Dziki, Danuta Sugier, Łukasz Pecio

**Affiliations:** 1 Department of Biochemistry and Food Chemistry, University of Life Sciences, 8 Skromna Str., 20-704 Lublin, Poland; 2 Department of Botany, Mycology and Ecology, Institute of Biological Sciences, Maria Curie-Skłodowska University, 19 Akademicka Str., 20-033 Lublin, Poland; piotr.sugier@poczta.umcs.lublin.pl; 3 Department of Thermal Technology and Food Process Engineering, University of Life Sciences in Lublin, 31 Głęboka St., 20-612 Lublin, Poland; dariusz.dziki@up.lublin.pl; 4 Department of Industrial and Medicinal Plants, University of Life Sciences in Lublin, 15 Akademicka Street, 20-950 Lublin, Poland; danuta.sugier@up.lublin.pl; 5 Department of Biochemistry and Crop Quality, Institute of Soil Science and Plant Cultivation State Research Institute, Czartoryskich Str. 8, 24-100 Pulawy, Poland; lpecio@iung.pulawy.pl

**Keywords:** *Stratiotes aloides* L., phenolics compounds, antioxidant activity, lipoxygenase inhibition, bioaccessibility

## Abstract

*Stratiotes aloides* L. is common water plant in central Poland. Due to its expansive character, *S. aloides* L. can strongly affect the functioning of aquatic ecosystems. *S. aloides* L. was an important famine plant in central Poland. This plant was commonly collected and cooked until the turn of the 20th century. It has also been used to heal wounds, especially when these are made by an iron implement. The objective of the present work was to study the phenolic profile in the leaves and roots of *S. aloides* as well as their antioxidant potential and ability to inhibit lipoxygenase (LOX) in the light of their potential bioaccessibility. The dominant compound in its leaves was luteolin-7-O-hexoside-glucuronide (5.84 mg/g DW), whereas the dominant root component was chrysoeriol-7-O-hexoside-glucuronide (0.83 mg/g DW). Infusions from leaves, roots, and their 1:1 (*v*/*v*) mixture contained potentially bioaccessible antiradical compounds. *S. aloides* is a good source of water-extractable reductive compounds. Especially valuable are the leaves of this plant. The roots of *S. aloides* contained very active hydrophilic compounds able to chelate metal ions. However, their potential bioaccessibility was relatively low. The hydrophilic compounds from the leaves were the most effective XO inhibitors (EC_50_ = 9.91 mg DW/mL). The water-extractable compounds derived from the leaves and roots acted as uncompetitive LOX inhibitors.

## 1. Introduction

Plants serve as an important source of natural products or chemical substances called phytochemicals. They are biologically active and play important roles in preventive and therapeutic medicines, protect plant cells from environmental hazards and pathogenic attacks, and contribute to their aroma and flavor. They can be accumulated in different parts of plants, and their concentration varies across different tissues and species [1]. 

Phytochemicals are a powerful group of compounds representing secondary metabolites of plants and including a diverse range of chemicals such as phenolic compounds, steroidal saponins, organosulphur compounds, and vitamins [2]. The multidirectional beneficial impact of phytochemicals on the human body is particularly important, thus investigations of a wide range of different plants as potential sources of antioxidants have become increasingly popular. At present, it is essential to preserve the biological balance in ecosystems; therefore, the use of invasive plants for this purpose seems particularly interesting. 

*Stratiotes aloides* L. (also called the water soldier) is now common in many water bodies where they often dominate. The water soldier plays a significant role in small aquatic ecosystems, being a rapid colonizer and a highly productive, most expansive aquatic macrophyte forming dense floating carpets. In Europe, a number of lakes and ponds throughout the UK have seen an invasion of the water soldier. *S. aloides* is indicated as a high impact (HI) species in the Norwegian Black List [3]. It occurs from east Europe through west Siberia and the Caucasus to northern Kazakhstan [4]. This plant is found in waters in the whole lowland area in Poland [5]. Due to its expansive character and high productivity, the plant may strongly affect the functioning of a water body and other macrophytes [6]. *S. aloides* is not usually regarded as a crop plant. Nonetheless, in northwestern parts of the former Soviet Union and in Poland, it is harvested both for fertilization of fields and market gardens and as a component in fodder for cattle or pigs, given the high phosphorus and potassium content of this green manure [7]. It appears to have a negligible history of use as human food; it was reported from a few locations in Poland immediately after World War II when near-famine conditions existed. Although it occurs over a large area of Poland, *S. aloides* was an important famine plant in central Poland, where it was commonly collected from the bottom of lakes and cooked until the turn of the 20th century [8]. 

As reported by [9], *S. aloides* is effective against St. Anthony’s Fire and alleviates swelling and inflammations in wounds. An ointment containing the plant has been found to heal wounds effectively. The author also reported its effective use to cure ‘bruised’ kidneys. In the past, it had the reputation of being an unfailing cure for all wounds made by iron weapons. Studies of the biological activity of *S. aloides* show that this plant can be used as a new potential source of active compounds with anti-inflammatory and anti-gout effects [10].

Therefore, the aim of this study was to examine the phenolic profile in the leaves and roots of *S. aloides* as well as their antioxidant potential and ability to inhibit lipoxygenase (LOX) in the light of their potential bioaccessibility. 

## 2. Results

The leaves of *S. aloides* were shown to be a rich source of flavonoid derivatives (especially luteolin and chrysoeriol). Luteolin-7-O-hexoside-glucuronide was the dominant compound in the leaves, whereas chrysoeriol-7-O-hexoside-glucuronide was the dominant root component (Table 1). 

**Figure 1:**
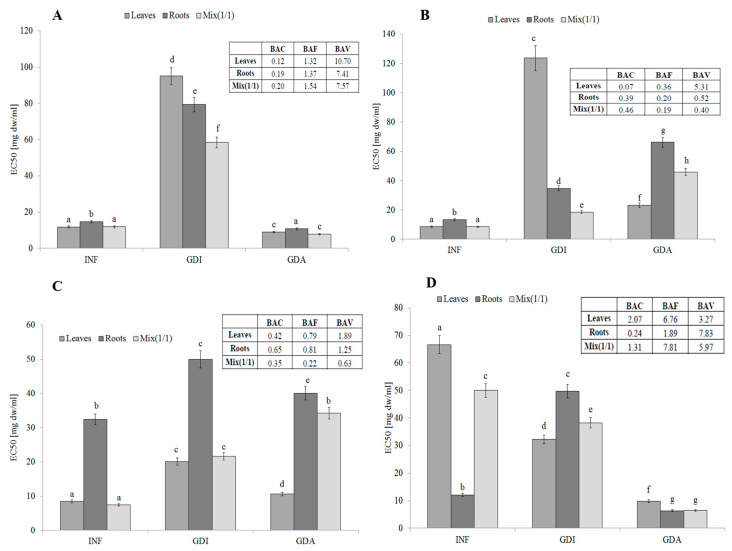
Antioxidant activity, potential bioaccessibility, bioavailability and bioefficiency of low-molecular antioxidants of leaves and roots of *S. aloides*. (**A**)—antiradical activity, (**B**)—ferric reducing power, (**C**)—inhibition of linoleic acid peroxidation, (**D**) metal chelating activity. INF—infusions, GDI—gastrointestinally digested extracts, GDA—samples after simulated absorption; BAC—the antioxidant bioaccessibility index, BAV—the antioxidant bioavailability index, BEF—the antioxidant bioefficiency index. Standard deviation (SD) is shown as error bars. Values (±SD) with different letters (a–h) are significantly different at *p* < 0.05.

The infusion from leaves, roots, and their mixture 1:1 (*v*/*v*) contained compounds with strong antiradical activity (measured against 2,2′-azino-bis(3-ethylbenzthiazoline-6-sulphonic acid; ABTS free radicals). The activity of leaves and all plant (MIX) was comparable (EC_50_ about 11.8 mg dw/mL). Unfortunately, the activity of potentially bioaccessible compounds from all samples was significantly lower. This tendency was especially visible in the case of the leaves and the mixture. The relatively high activity of the mixture may suggest synergistic action between compounds derived from the leaves and roots. Most importantly, the highest activity was determined for samples obtained after simulated absorption, which may suggest their high bioavailability (Figure 1A). The BAC (antioxidant bioaccessibility index) value clearly indicated that the compounds present in the infusions were poorly bioaccessible in vitro. The lowest bioaccessibility was determined for the roots and the mixture (BAC values of 0.18 and 0.20, respectively). Most importantly, the free radical scavengers released during simulated digestion were highly bioavailable in vitro. This fact was confirmed by the BAV (antioxidant bioavailability index) values (10.69 for the leaves and approx. 7.5 for the roots and mixture). Most importantly, the BAF (antioxidant bioefficiency index) values indicated that compounds that were able to permeate the dialysis membrane were more active (ca. 30% for the leaves and roots and 54% for the mixture) than compounds present in the initial infusions (Figure 1A).

*S. aloides* is a good source of water-extractable compounds with strong reducing potential. Especially valuable were the leaves of this plant. Unfortunately, the conditions prevailing in the simulated gastrointestinal system resulted in a significant decrease in their activity, especially in the case of the leaves. Additionally, potential synergism between compounds contained in the leaves and roots was observed in this case. Potentially bioavailable compounds demonstrated significantly higher activity (compared to the gastrointestinally digested samples, GDI), which may suggest their potential nutraceutical value (Figure 1B). The activity of the reductive phytochemicals present in the infusions drastically declined after the simulated digestion. The lowest BAC value (0.07) was determined in the case of the leaf extract. However, the active compounds were able to permeate the dialysis membrane, which suggests their high bioavailability (BAV value of approx. 5.3). Unfortunately, the reductive compounds derived from the roots and the mix were poorly bioavailable in vitro. In general, the simulated digestion and absorption caused a significant decrease in the reducing power of all samples (compared to the infusions), which points to their poor bioavailability, not exceeding 40% (BAF values from 0.36 for the leaves to 0.18 for the mix) (Figure 1B).

Interesting results were obtained by determination of the ability to protect lipids against oxidation (LPO). The leaves of *S. aloides* were the best source of compounds with this activity. Importantly, the compounds contained in the leaves were potentially bioaccessible and bioavailable. Based on these results, it can be assumed that the water-extractable and potentially bioaccessible active phytochemicals from the leaves and roots acted synergistically (Figure 1C). The BAC values indicated quite poor bioaccessibility of the lipid protectors, especially in the case of the mix (0.35). The BAV index analysis suggested that the active compounds released during the simulated digestion from the leaves and roots were bioavailable in vitro (BAV values of 1.89 and 1.24, respectively); in turn, poor bioavailability was determined in the case of MIX (BAV = 0.63). Compared to the initial infusions, relatively low bioavailability was determined for the leaves and roots (BAF value of ca. 0.8), while the lowest BAF value was found in the case of MIX (Figure 1C).

In contrast to LPO, the roots of *S. aloides* contained very active hydrophilic compounds able to chelate metal ions. Unfortunately, their potential bioaccessibility was relatively low, probably due to interactions with other compounds released from the matrix (e.g., proteins and/or starch). Most importantly, the active compounds were highly bioavailable in vitro, which confirms their hydrophilicity (Figure 1D). The leaves from the analyzed plant were also a good source of potentially bioavailable compounds with strong chelating power. Importantly, the compounds with the ability to protect lipids contained in the leaves seemed to be highly bioaccessible and bioavailable in vitro (BAC value of ca. 2.07; BAF value of 6.76). The BAV index indicates that compounds released during simulated gastrointestinal digestion interact with active components, probably creating inactive combinations (complexes); however, this activity increased significantly after dialysis (simulating passive absorption). This is probably related to the fact that large molecules (matrix components) are unable to penetrate through the semi-permeable membrane. This situation is clearly visible in the case of the roots (Figure 1D). 

**Figure 2:**
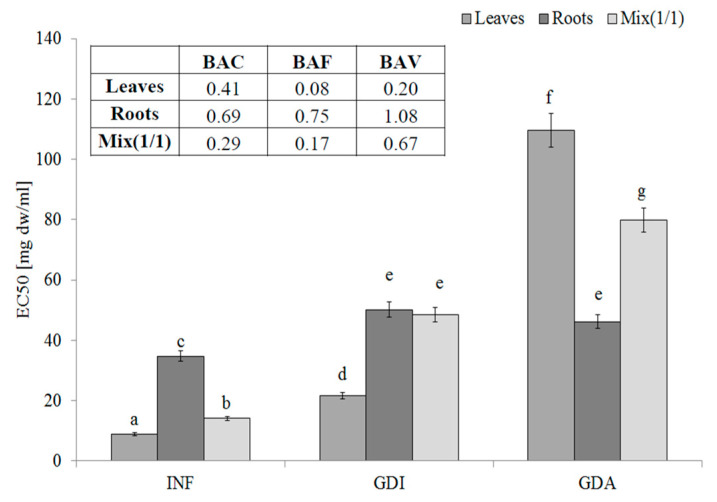
Xanthine oxidase-inhibitory potential of extracts from the leaves and roots of *S. aloides*.

One of most effective endogenous sources of free radicals is xanthine oxidase (XO). The hydrophilic compounds from the leaves were the most effective XO inhibitors (EC_50_ = 8.91 mg DW/mL). Unfortunately, their activity decreased during gastrointestinal digestion and the active compounds seemed to be poorly bioavailable. These observations were confirmed by the low BAC index value (0.41, 0.69, and 0.29 for the leaves, roots, and mixture, respectively). Most importantly, compounds with an ability to inhibit XO activity were poorly bioavailable in vitro (see the BAF values) and in this case, the interactions with other matrix components (released during the in vitro digestion) had a rather positive effect, especially in the case of the leaves and MIX (Figure 2).

INF—infusions, GDI—gastrointestinally digested extracts, GDA—samples after simulated absorption; BAC—the antioxidant bioaccessibility index, BAV—the antioxidant bioavailability index, BEF—the antioxidant bioefficiency index. Standard deviation (SD) is shown as error bars. Values (±SD) with different letters (a–g) are significantly different at *p* < 0.05.

**Figure 3:**
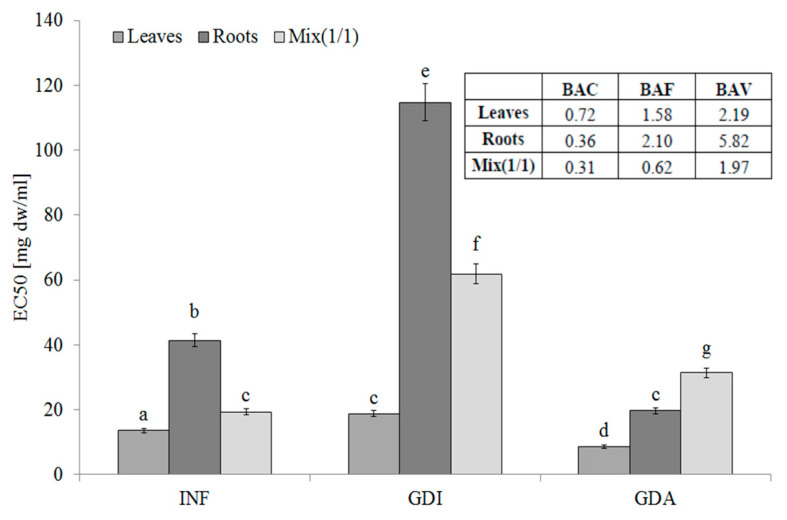
Lipoxygenase-inhibitory potential of extracts from the leaves and roots of *S. aloides*. INF —infusions, GDI—gastrointestinally digested extracts, GDA—samples after simulated absorption; BAC—the antioxidant bioaccessibility index, BAV—the antioxidant bioavailability index, BEF—the antioxidant bioefficiency index. Standard deviation (SD) is shown as error bars. Values (±SD) with different letters (a–g) are significantly different at *p* < 0.05.

The leaves of *S. aloides* contained hydrophilic compounds with an ability to inhibit LOX, whereas the lowest activity was determined for the root infusions. Additionally, the phytochemicals from the roots were poorly bioaccessible in vitro. Most importantly, the LOX inhibitors from the leaves and roots were highly bioavailable in vitro. This fact was confirmed by the BAF values. It should be underlined that, as indicated by the BAV index values, compounds with high molecular weight interact negatively with LOX inhibitors. They seem to be low molecular weight compounds, as they were able to permeate the dialysis membrane (see BAV values) (Figure 3). 

**Figure 4:**
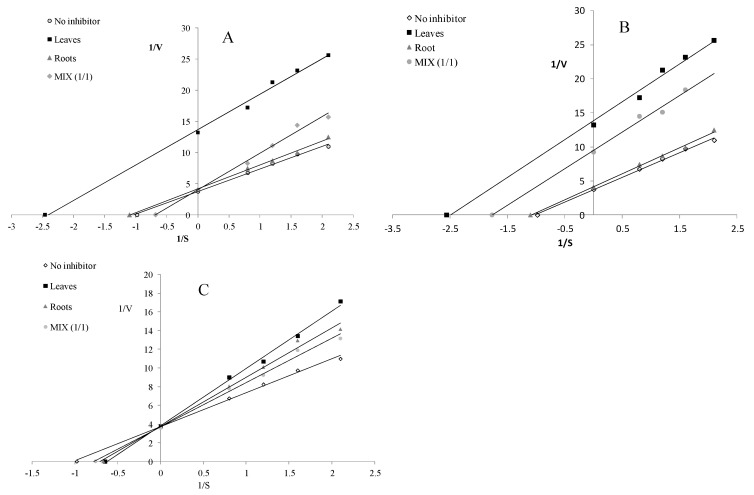
Mode of lipoxygenase inhibition by extracts from the leaves and roots of *S. aloides*. (**A**)—infusions, (**B**)—extracts after digestion in vitro, (**C**)—extracts after absorption in vitro.

The water-extractable compounds derived from the leaves and roots acted as uncompetitive LOX inhibitors, whereas the infusion from MIX inhibited LOX in a competitive manner (Figure 4A). Compounds released during the simulated gastrointestinal digestion acted as uncompetitive LOX inhibitors (Figure 4B), whereas the potentially bioavailable compounds demonstrated a competitive mode of action (Figure 4C). 

Importantly, as demonstrated by our research, the whole plant exhibits activity and can therefore be used in its entirety without the cumbersome and time-consuming separation of leaves from roots. The interaction factors (IF) coefficient reflecting the interactions between the biologically active components of the extracts was determined to confirm this thesis. It was found that the water-extractable compounds with an ability to inhibit XO activity and protect lipids against oxidation acted synergistically. Moderate synergism was found in the case of the LOX inhibitors and reductive compounds. The antiradical phytochemicals acted nearly additively, whereas moderate antagonism was determined for the metal ion chelating compounds (Table 2). The conditions prevailing in the simulated gastrointestinal system changed the interactions between the biologically active compounds contained in *S. aloides*. Strong synergism was determined for the potentially bioaccessible compounds with a reducing capacity, while phytochemicals protecting lipids against oxidation and LPO and scavenging free ABTS radicals acted synergistically. In turn, nearly additive interactions were determined for the potentially bioaccessible compounds with an ability to chelate metal ions and for the LOX inhibitors. Moderate antagonism was found only in the case of XO inhibitors. The samples after simulated absorption (GDA) extracts contained small hydrophilic molecules able to permeate the dialysis membrane. Among them, antiradical compounds and those with chelating power acted synergistically. Taking into account the potentially bioavailable compounds, moderate synergism was found for the antiradical and chelating compounds. Phytochemicals with reducing power and XO acted additively. Antagonism was determined for the LOX inhibitors, whereas moderate antagonism was found for compounds that were able to protect lipids against oxidation (Table 2).

## 3. Discussion

The first phytochemical analysis of the aquatic macrophyte *S. aloides* conducted by [11] revealed the presence of two new flavonoid glucuronides: luteolin 7-O-D-glucopyranosiduronic acid-(1f2)-D-glucopyranoside and chrysoeriol 7-O-D-glucopyranosiduronic acid-(1f2)-D-glucopyranoside. Additionally, new compounds 2-(2-hydroxypentyl)-5-carboxy-7-methoxychromone, chrysoeriol 7-O-(6-O-malonyl) glucopyranoside, and a flavonoid glycoside luteolin 7-O-(6-O-malonyl)glucopyranoside were isolated. Recent investigations have shown that antioxidants of plant origin may have great importance as therapeutic agents in several diseases caused by oxidative stress [12] Many synthetic antioxidant compounds have shown toxic and/or mutagenic effects, which have stimulated the interest of many investigators to search for natural antioxidants [13]. According to WHO, 80% of the world’s population are estimated to use some form of herbal medicine in their health care. It was also reported that approximately one quarter of prescribed drugs contain plant extracts or active ingredients obtained from plants. To prove this, a number of researchers have done extensive work on various plants [14]. The multidirectional antioxidant effect of *S. aloides* extracts is the result of their unique composition. As presented in Table 1, both the leaves and roots of *S. aloides* contain a large amount of flavonoids, especially luteolin, apigenin, and chrysoeriol derivatives. Flavonoids are the largest group of naturally occurring polyphenols characterized by broad in vitro and in vivo biological activities. These compounds act as multidirectional antioxidants exerting anti-mutagenic, anti-inflammatory, and antiviral effects. Most flavonoids are potent metal chelators and possess strong antioxidant and free radical scavenging activity [15]. They are also known to be potent inhibitors for several enzymes, such as xanthine oxidase, cyclooxygenase, and lipoxygenase [16]. Luteolin exerts specific anti-inflammatory and anti-carcinogenic effects connected with its antioxidant and free radical scavenging capacities. It was found to inhibit tumor cell proliferation with IC_50_ values between 3 and 50 μM in vitro and in vivo administered at the dose from 5 to 10 mg/kg i.p., via intragastric application of 0.1–0.3 mg/kg/d, or as a food additive at concentrations from 50 to 200 ppm. It was effective in 10 cancer types, with the lowest IC_50_ for carcinoma of the stomach (25 μM), cervix (27 μM), lung (41 μM), and bladder (68 μM) [17]. Luteolin has received increasing attention due to its various biological activities and pharmacological effects and has been widely applied in medicine and production of functional foods [15].

Apigenin is one of the most widely distributed flavonoids in the plant kingdom. It is present principally in a glycosylated form in a significant amount of vegetables, fruits, herbs, and plant-based beverages. The two main commonly known sources of apigenin are parsley (*Petroselinum crispum*) and peppers (*Piper nigrum*) containing 13.526 mg/100 g and 4.98 mg/100 g of the compound, respectively [18]. The data presented in Table 1 show that the leaves and roots of *S. aloides* are a very rich source of this compound (0.219 and 0.071 mg/g dw, respectively). 

Chrysoeriol, i.e., a flavonoid found mainly in tropical plants, deserves special mention. The presence of derivatives of this compound has also been shown in celery leaves and seeds [19]. However, there are only a few reports in the literature on its antioxidant activity [20]. Chrysoeriol isolated from peanut hulls inhibited lipid peroxidation in low-density lipoprotein induced by Cu^2+/^O_2_ with an IC_50_ value of 2.6 mM. Chrysoeriol isolated from the aerial part of the gaiyou exhibited antimutagenic activity in *Salmonella typhimurium* TA98 [21]. In turn, chrysoeriol isolated from *Morinda morindoides* leaves has been found to be ineffective towards superoxide radicals generated from xanthine and xanthine oxidase [21].

It has been demonstrated that XO inhibitors are potential therapeutic agents for hyperuricemia that causes gout, renal stones, myocardial ischemia, and ROS-induced diseases. Regrettably, all these medications (including allopurinol, i.e., a potent XO inhibitor clinically used for gout treatment) are associated with some side effects such as skin rashes, hepatitis, fever, Stevens–Johnson syndrome, nephropathy, fatal liver necrosis, and allergic reactions [22]. As shown by [16], apigenin had a comparable inhibitory effect to that of allopurinol. Genistein was a weaker inhibitor than quercetin and myricetin with a Ki value of 3.23 μM. Results obtained by Dong et al. indicated that luteolin and a luteolin–manganese(II) complex acted as competitive XO inhibitors with good radical scavenging activity and antioxidant activities [15]. As reported by Nguyen et al., luteolin, apigenin, and chrysoeriol derived from the *Chrysanthemum sinese* flower exhibited strong XO inhibitory activities (IC_50_: 1.24, 0.13, and 0.20 μM, respectively) [23]. 

Several reports have indicated that flavonoids were able to inhibit XO activity [16]. The results presented in this paper confirm this thesis, also pointing to the role of potential interactions between active compounds in such a complex system as a plant extract.

Recently, many of the studied plant polyphenols have been tested for their antioxidant potential [24]. Thus, the content and activity of low-molecular antioxidants may play a crucial role in the pro-health properties of plant-derived food; however, equally important is the limitation of the generation of ROS by endogenous factors, which include e.g., lipoxygenases (LOXs) [25]. 

LOXs are dioxygenases catalyzing the formation of corresponding hydroperoxides from polyunsaturated fatty acids such as linoleic and arachidonic acids. LOX enzymes are expressed in immune, epithelial, and tumor cells that display a variety of physiological functions, including inflammation, skin disorder, and tumorigenesis [26]. Because human lipoxygenase isoenzymes (5-, 12-, and 15-lipoxygenases) are extremely unstable at normal physiological temperatures in the cell-free lipoxygenase assay, the physiological temperature-stable and commercially available plant LOX derived from *Glycine max* is an appropriate model for the LOX inhibitory assay [27].

LOX is not only involved in oxidation of lipids but also production of leukotriene, which mediates the occurrence of inflammation, which is painful and can lead to diseases that pose a threat to public health. Inhibition of LOX activity is a prospective method for treatment of inflammation; hence, many specific compounds have been designed and synthesized as LOX inhibitors [28].

There are mainly two types of anti-leukotriene drugs, i.e., cysteinyl leukotriene receptor antagonists and leukotriene synthesis inhibitors (5-LOX inhibitors): zileuton, ZD-2138, BayX 1005, and MK-0591. Treatment with oral montelukast, zafirlukast, or zileuton significantly improves many clinical outcomes. Given the danger of liver disruption, zileuton is not commonly used (it is unlicensed in Poland) [29]. The ability of extracts from vegetables, medicinal plants, some spices, and cereals to inhibit LOX has already been studied [25,27,30]; however, in the recent literature, there is no information about LOX inhibition by *S. aloides* components. The study of the interaction of flavonoids with LOX is of great interest, as this enzyme may be considered a target for the health beneficial effect of flavonoids. It is known that mammalian LOX is inhibited by flavan-3-ols and that low-density lipoprotein oxidation induced by 15-LOX is inhibited by quercetin and epicatechin. It has also been reported that epicatechin and its related oligomers, i.e., procyanidins, inhibit human 5-LOX contributing to an anti-inflammatory effect [31]. 

## 4. Materials and Methods 

### 4.1. Chemicals

Ferrozine (3-(2-pyridyl)-5,6-bis-(4-phenyl-sulfonic acid)-1,2,4-triazine), ABTS (2,2′-azino-bis(3-ethylbenzthiazoline-6-sulphonic acid)), α-amylase, pancreatin, pepsin, xanthine oxidase, lipoxygenase (from *Glycine max*, type 1_B), bile extract, Folin-Ciocalteau reagent, linoleic acid and ammonium thiocyanate were purchased from Sigma-Aldrich company (Poznan, Poland). All others chemicals were of analytical grade.

### 4.2. Plant Material

Plants of *S. aloides* were collected from the Łęczna-Włodawa Lake District located in mid-eastern Poland (51°17′49″ N and 51°35′56″ N; 22°50′36″ E and 23°42′17″ E) in 2017. Plants were dried at 45 °C and ground before extraction.

### 4.3. Phenolic Profile Analysis (Ultra-Performance Liquid Chromatography – Mass Spectrometry)

Sample Preparation. Extraction was performed on a Dionex ASE 200 extractor (Dionex Corp., Sunnyvale, CA, USA). A portion of 100 mg of the dried and ground sample was mixed with diatomaceous earth (Dionex ASE Prep DE, Dionex Corp., Sunnyvale, CA, USA) and placed in a 5 mL stainless steel extraction cell. A cellulose filter (Dionex Corp.) together with 1 g of LiChroprep RP-18 (40–63 µm) (Merck, Darmstadt, Germany) was placed at the bottom of the extraction cell to facilitate chlorophyll removal. Three static extraction cycles, 5 min each, were applied—first, the cells were filled with extraction solvent (80% methanol in water, *v*/*v*), pressurized at 1500 psi, and heated at 120 °C for 5 min to ensure that samples reached thermal equilibrium. After extraction, cells were rinsed with fresh solvent (50% of the cell volume) and purged with a flow of nitrogen for 100 s. The extracts (25 mL each) were collected into the 40 mL glass vials, evaporated to dryness using a rotary evaporator at 40 °C, suspended in 1 mL of 80% methanol, and stored at −20 °C until required. Phenolic constituents of *S. aloides* were analyzed using a Waters ACQUITY UPLC system (Waters Corp., Milford, MA, USA), comprising of a binary pump system, sample manager, column manager, and PDA detector (Waters Corp., Milford, MA, USA). The acquisition and data processing were performed using Waters MassLynx software v.4.1. The samples were chromatographed on a BEH C18 column (100 mm × 2.1 mm i.d., 1.7 μm, Waters Corp., Milford, MA, USA), which was kept at 40 °C. The flow rate was adjusted to 400 µL min^−1^. The following solvent system: mobile phase A (0.1% formic acid in Milli-Q water, *v*/*v*) and mobile phase B (0.1 % formic acid in MeCN, *v*/*v*) was applied. The gradient program was as follows: 0–1.0 min, 5% B; 1.0–24.0 min, 5–50% B; 24.0–25.0 min, 50–95% B; 25.0–27.0 min, 95% B; 27.0–27.1 min, 95-5% B; 27.1–30.0 min, 5% B. Samples were thermostatted at 8 °C in the sample manager. The injection volume of the sample was 2.5 μL (partial loop with needle overfill mode) and samples were analyzed in triplicate. Strong needle wash solution (1:1:1, MeOH-MeCN-iPrOH, *v*/*v*/*v*) and weak needle wash solution (5:95, MeCN–H_2_O, *v*/*v*) were used. The detection wavelength was set at 255 nm (3.6 nm resolution) for the quantification of phenolics, at a 5 points s-1 rate. The separation was completed in 30 min. Peaks were assigned based on their UV spectra, mass to charge ratio (*m*/*z*), and ESI-MS/MS fragmentation patterns. Rutin (quercetin rutinoside) was used as a group standard for determination of phenolic compounds. The calibration curve was constructed with 7 points in the range of 5.2–419.5 µM, and the concentration of constituents in plant material was then calculated based on molecular weights read from the MS spectra. The MS analyses were carried out on a TQD mass spectrometer (Waters Corp., Milford, MA, USA) equipped with a Z-spray electrospray interface. The following instrumental parameters were used for ESI-MS analysis of phenolic compounds (negative ionization mode): capillary voltage, 2.8 kV; cone voltage, 45 V; desolvation gas, N2 800 L h^−1^; cone gas, N2 100 L h^−1^; source temp. 140 °C, desolvation temp. 350 °C. Compounds were analyzed in full scan mode (mass range of *m*/*z* 200–1200 was scanned).

### 4.4. Extracts Preparations

To make extracts, the plant was divided into individual parts, i.e., root and leaves, dried and then powdered. Aqueous solutions were prepared by extracting 0.2 g of the sample in 20 mL boiling deionized water. After cooling, the samples were centrifuged. Supernatants (INF) were intended for further research.

### 4.5. In vitro Digestion

In vitro digestion of plant materials was performed according to [32] with some modifications. Fluids obtained after simulated gastrointestinal digestion (GDI) were treated as the equivalent of a potentially bioaccessible fraction. GDI fluid was transferred to dialysis sacks (D9777-100FT, Sigma-Aldrich, Poznan, Poland), placed in an Erlenmeyer flask containing 50 mL of PBS buffer, and incubated in a rotary shaker (twice per 2 h, 37 °C). The PBS buffer, together with the compounds that passed through the membrane (dialysate, GDA), was treated as an equivalent of the raw material absorbed in the intestines after digestion (potentially bioavailable fraction).

### 4.6. Antioxidant Assay

#### 4.6.1. Ability to Quench ABTS Radicals

The ABTS radical scavenging activity was determined according to [33], with some modifications. The ability of samples to quench the ABTS free radical was assessed according to the following equation:

scavenging% = [(A_C_ − A_A_)/A_C_)] × 100, (1)

where A_C_ is the absorbance of the control and A_A_ is the absorbance of the sample.

The half maximal inhibitory concentration EC_50_ value was determined by interpolation of the dose–response curves. The EC_50_ values were calculated at fitted models as the concentration of the tested compound gave 50% of the maximum inhibition based on a dose-dependent mode of action.

#### 4.6.2. Ferric Reducing Power (FRAP)

Reducing power was determined according to [34]. 500 μL of 200 mM phosphate buffered saline extract and 500 μL of a 1% solution of potassium ferricyanide were added to 500 μL of the extract. The prepared mixture was insulated at 50 °C for 20 min. Then, 500 μL of 10% trichloroacetic acid (TCA) was added and the mixture was rested for few minutes. 1 mL of that mixture was taken and mixed with 1 mL of deionized water and 0.2 mL of 1% iron (II) chloride. Absorbance was measured at 725 nm. Reducing power determined as EC_50_ is the effective concentration at which the absorbance was 0.5 for reducing power and was obtained by interpolation from linear regression analysis.

#### 4.6.3. Metal Chelating Activity (CHP)

Chelating power was determined by the method of [35]. The chelating potential was assessed according to the formula:

% inhibition = [1 − (As/Ac)] × 100,(2)

where Ac is the absorbance of the control and As is the absorbance of the sample.

Antioxidant activity was determined as EC_50_ – extract concentration providing 50% of activity was based on a dose-dependent mode of action.

#### 4.6.4. Inhibition of Linoleic Acid Peroxidation

Antioxidant activity was determined as the degree of inhibition of the peroxidation of linoleic acid according to [36] with the following modification: instead of hemoglobin, 10 mmol/L FeCl_2_ in water was used. All measurements were performed in four replicates. Antioxidant activity was determined as EC_50_—extract concentration providing 50% of activity was based on a dose-dependent mode of action.

#### 4.6.5. Determination of Xanthine Oxidase—Inhibitory (XOI) Activity

The XOI activities with xanthine as a substrate were estimated according to [37] with modification. Inhibitory activity was expressed as EC_50_ (efficient concentration): the amount of sample; mg of dry weight (DW) needed to obtain 50% activity per 1.0 mL of the initial solution.

#### 4.6.6. Inhibition of Lipoxygenase Activity (LOXI)

Lipoxygenase activity was determined according to method described by [38] adapted for microplate reader (Epoch 2 Microplate Spectrophotometer, BioTek Instruments, Winooski, Vermont, USA) [39]. One unit of LOX activity was defined as an increase in absorbance of 0.001 per minute at 234 nm. Antioxidant activities were determined as EC_50_—extract concentration provided 50% of activity was based on dose-dependent mode of action.

### 4.7. Theoretical Approaches

For better evaluation bioaccessibility and bioavailability in vitro the following factors were determined [40]:

-he antioxidant bioaccessibility index (BAC), which is an indication of the bioaccessibility of antioxidative compounds:

BAC = A_INF_/A_GDI_, (3)

-the antioxidant bioavailability index (BAV), which is an indication of the bioavailability of antioxidative compounds:

BAV = A_GDI_/A_GDA_, (4)

- the antioxidant bioefficiency index (BAF), which is an indication of the bioactivity of bioavailable antioxidant compounds:

BAF = A_INF_/A_GDA_, (5)

where, A_INF_ is EC_50_ of infusion (INF), A_GDI_ is EC_50_ of extract after simulated gastrointestinal digestion (GDI), A_GDA_ is EC_50_ of extract after simulated intestinal absorption (GDA), 

For estimation of interaction of compounds contained in leaves and roots of *S. aloides* the interaction factor (IF) was determined:

IF = A_M_/A_T_, (6)

where, A_M_ = the measured activity of a mixture of samples, and A_T_ = the theoretically calculated mixture activity (based on the dose response of single components at various concentrations).

IF value < 1 indicates synergistic interaction; IF > 1 indicates antagonism; IF ≈ 1 indicates additional interactions.

### 4.8. Statistical Analysis 

Experimental data were shown as means ± S.D for biochemical assays, statistical significance was estimated through Turkey’s test for the data obtained from three independent samples of each extract in three parallel experiments (n = 9). Statistical tests were carried out at a significance level of α = 0.05. Statistical tests were performed using Statistica 6.0 software (StatSoft, Inc., Tulsa, OK, USA).

## 5. Conclusions

*S. aloides* is an invasive plant posing a problem for aquatic ecosystems. Our results indicate that *S. aloides* is a good source of flavonoid derivatives exhibiting strong multidirectional antioxidant activity. In addition, the extracts from *S. aloides* exhibited an ability to inhibit LOX activity, which suggests their anti-inflammatory potential. This, in combination with the high ability to chelate metal ions, may confirm the usefulness of *S. aloides* in the treatment of wounds caused by iron weapons. Most importantly, the active compounds were potentially bioaccessible and bioavailable. The results indicate a possibility of using this plant to produce preparations with health-promoting properties (nutraceuticals, functional food supplements). To the best of our knowledge, this is the first report of the evaluation of the antioxidant capacity as well as lipoxygenase inhibitory activity of potentially bioaccessible and bioavailable components of *S. aloides*.

## Figures and Tables

**Table 1:** Qualitative and quantitative UPLC-MS/MS analysis of phenolics from the leaves and roots of *Stratiotes aloides* (*n = 9*).

Compound		Concentration ± SD [mg g^−1^]	
		*Stratiotes aloides*	
		Leaves	Roots
1	luteolin-7-O-hexoside-glucuronide	5.838 ± 0.440 *^a^	0.128 ± 0.008 ^b^
2	apigenin-7-O-hexoside-glucuronide	0.219 ± 0.004 ^e^	0.071 ± 0.010 ^c^
3	luteolin-7-O-hexoside (*isomer I*)	2.652 ± 0.087 ^c^	0.022 ± 0.003 ^d^
4	chrysoeriol-7-O-hexoside-glucuronide (*isomer I*)	3.375 ± 0.496 ^b^	0.831 ± 0.062 ^a^
5	luteolin-7-O-hexoside (*isomer II*)	0.290 ± 0.008 ^d^	nd
6	luteolin-7-O-malonyl-hexoside	0.209 ± 0.004 ^e^	0.017 ± 0.001 ^f^
7	chrysoeriol-7-O-hexoside (*isomer I*)	0.299 ± 0.008 ^d^	0.044 ± 0.004 ^d^
8	chrysoeriol-7-O-hexoside (*isomer II*)	0.071 ± 0.004 ^f^	nd
9	luteolin	0.062 ± 0.002 ^f^	nd
10	chrysoeriol	0.039 ± 0.000 ^g^	0.016 ± 0.001 ^f^
	sum	13.053 ± 0.955	1.129 ± 0.078

* data represent means ± SD. Means (n = 9) followed by the different lowercase letters ^(a–g)^ in columns are significantly different at *p* < 0.05.; nd—not detected.

**Table 2:** Comparison of theoretical activity (At), measured activity (Am)and interaction factors (IF) of extracts from leaves and root of *S. aloides.*

Activity	INF			GDI			GDA		
	At *	Am	IF	At	Am	IF	At	Am	IF
CHEL	39.34	50.00	1.27	40.97	38.21	0.93	8.10	6.40	0.79
FRAP	10.96	8.52	0.78	79.17	18.48	0.23	44.79	45.83	1.02
LPO	20.42	7.47	0.37	35.04	21.61	0.62	25.33	34.18	1.35
ABTS	13.23	11.88	0.90	87.19	58.44	0.67	9.80	7.72	0.79
LOX	27.47	19.36	0.70	66.76	61.85	0.93	14.15	31.34	2.22
XO	21.79	14.12	0.65	35.87	48.42	1.35	77.91	79.75	1.02

* expressed as EC50 [mg dw/mL]; INF- infusion, GDI—samples after gastrointestinal digestion, GDA—samples after simulated absorption, CHEL—chelating power, FRAP—ferric reducing power, LPO—ability to protect lipids against oxidation, ABTS—antiradical potential, LOX—ability to lipoxygenase inhibition, XO—ability to xanthine oxidase inhibition.
